# The Clinical Impact of Platelets on Post-Injury Serum Creatinine Concentration in Multiple Trauma Patients: A Retrospective Cohort Study

**DOI:** 10.3390/medicina58070901

**Published:** 2022-07-06

**Authors:** Frederik Greve, Ina Aulbach, Olivia Mair, Peter Biberthaler, Marc Hanschen

**Affiliations:** 1 Department of Trauma Surgery, Klinikum rechts der Isar, Technical University of Munich, 81675 Munich, Germany; ina.aulbach@charite.de (I.A.); oliviaanna.mair@mri.tum.de (O.M.); peter.biberthaler@mri.tum.de (P.B.); marc.hanschen@mri.tum.de (M.H.); 2 Department of Traumatology and Reconstructive Surgery, Charité-Universitätsmedizin Berlin, 12203 Berlin, Germany

**Keywords:** platelets, creatinine, multiple trauma, immune system, hyperinflammation, post-traumatic organ failure

## Abstract

*Background and objective*: Platelets contribute to the immunological response after multiple trauma. To determine the clinical impact, this study analyzes the association between platelets and creatinine concentration as an indicator of kidney function in polytraumatized patients. *Methods:* We investigated all patients presenting an Injury Severity Score (ISS) ≥16 for a 2-year period at our trauma center. Platelet counts and creatinine concentrations were analyzed, and correlation analysis was performed within 10 days after multiple trauma. *Results*: 83 patients with a median ISS of 22 were included. Platelet count was decreased on day 3 (*p* ≤ 0.001) and increased on day 10 (*p* ≤ 0.001). Platelet count was elevated on day 10 in younger patients and diminished in severely injured patients (ISS ≥35) on day 1 (*p* = 0.012) and day 3 (*p* = 0.011). Creatinine concentration was decreased on day 1 (*p* = 0.003) and day 10 (*p* ≤ 0.001) in female patients. Age (*p* = 0.01), male sex (*p* = 0.004), and injury severity (*p* = 0.014) were identified as factors for increased creatinine concentration on day 1, whereas platelets (*p* = 0.046) were associated with decreased creatinine concentrations on day 5 after multiple trauma. *Conclusions*: Kinetics of platelet count and creatinine concentration are influenced by age, gender, and trauma severity. There was no clear correlation between platelet counts and creatinine concentration. However, platelets seem to have a modulating effect on creatinine concentrations in the vulnerable phase after trauma.

## 1. Introduction

Trauma-induced injury remains among the most common causes of death worldwide [1]. Post-traumatic hyperinflammation, which leaves the patient vulnerable after initial damage control surgery, is a challenging aspect of treating multiple trauma patients. Unbalance and high intensity of the systematic inflammatory response syndrome (SIRS) and compensatory anti-inflammatory response syndrome (CARS) trigger complex pathophysiological cascades leading to organ dysfunction and multiple organ failure (MOF) as a potential lethal consequence [2,3,4,5,6,7,8].

The role of platelets in post-traumatic immune disturbance has gained increasing interest. Platelets contribute to hyperinflammation mainly by releasing proinflammatory mediators and heterogenous interactions with leukocytes [9,10,11,12,13,14,15,16,17,18].

Acute kidney injury (AKI) is frequently encountered in the context of hyperinflammation in the intensive care unit (ICU) setting and is associated with increased morbidity and mortality [8,19,20,21,22,23]. Platelets contribute to AKI by forming platelet-leukocyte aggregates, which impair endothelial integrity and lead to an obstruction of flow in the peritubular capillaries [24,25,26,27,28]. However, the exact pathophysiological mechanisms are still unclear. The majority of current research regarding platelet pathophysiology derives from experimental studies [14,29,30,31]. Few trials highlight the impact of post-traumatic platelet counts in the clinical setting, mainly limited to the development of acute respiratory distress syndrome (ARDS) [32,33,34,35,36]. Despite AKI being a significant component of MOF, there is no clinical study directly investigating the impact of platelet counts on kidney physiology in a post-traumatic setting.

We recently investigated the influence of platelet counts on post-traumatic lung physiology within the first 72 h after trauma [37]. We demonstrated a positive correlation between platelet counts and PaO_2_/FiO_2_-Index in only severely injured patients presenting an injury severity score (ISS) ≥ 35. Furthermore, the dynamics of platelet counts were influenced by trauma severity, whereas sex and age did not show any modulating effects.

As a complement to our previous work, this study focuses on the correlation between platelet counts and post-traumatic kidney physiology. The findings in this report provide a foundation for additional studies and could be utilized to objectively stratify patients at risk for MOF development based on objective data.

## 2. Materials and Methods

This single-center retrospective cohort study analyzes the dynamics and correlation of platelets and serum creatinine concentration as a surrogate marker of end-organ failure of the kidney in multiple trauma patients.

Due to previous identification as relevant influencing epidemiological factors for the development of post-traumatic MOF, we divided our study group into three subgroups according to age, gender, and injury severity [33,38,39,40].

Patients admitted for multiple trauma between 2015 and 2016 with an ISS of at least 16 were included. Patients with incomplete data files were excluded from the study.

Ethical approval was obtained from the local ethics committee. This investigation is additionally listed in the German Clinical Trial Register (www.drks.de, trial number: DRKS00025982, accessed on 25 March 2022) and linked to the International Clinical Trials Registry Platform of the World Health Organization (https://trialsearch.who.int, accessed on 25 March 2022).

### 2.1. Descriptive Analysis

Data of the included patients were analyzed for general and demographic information, injury characteristics, need for intensive care treatment, and survival. The Abbreviated Injury Scale (AIS) and ISS were used to describe trauma severity [41,42].

Platelet counts and serum creatinine concentration were assessed on several days after trauma (day 1, day 3, day 5, and day 10 after admission). In variance with our previous study [37], the observation period was extended and analyzed dynamic changes in post-traumatic platelet counts and serum creatinine concentration until day 10 after admission.

For the assessment of dynamic changes over time, analysis for repeated measures (mixed-effects model) was performed with the entire study population. Platelet counts and serum creatinine concentrations on the respective post-traumatic days were compared to day 1 as baseline values using Tukey’s multiple comparison test. Normal distribution was tested using the D’Agostino and Pearson test. Normally distributed values are presented as mean and standard deviation (SD). Non-normally distributed data are presented as the median and interquartile range (IQR). Statistical testing between two groups within the subgroups (<60 years vs. ≥60 years, male vs. female, and ISS < 35 vs. ISS ≥ 35) was performed by using the Mann-Whitney *U* test (non-normally distributed data) and *t*-test (normally distributed data).

### 2.2. Correlation Analysis

For correlation between platelet counts and serum creatinine concentration, correlation coefficients (Pearson/Spearman’s in dependence of data distribution) were determined for each time point (days 1, 3, 5, and 10).

For additive investigation of the influence of age, gender, and injury severity, multiple least-squares linear regression analysis with creatinine concentration as a dependent variable was conducted.

The level of significance was set as *p* < 0.05. Statistical testing was performed using GraphPad PRISM Software (San Diego, CA, USA).

## 3. Results

### 3.1. Descriptive Analysis

#### 3.1.1. Description of the Study Population

Data of 189 multiple trauma patients admitted to our hospital were screened for inclusion. The screening process rendered 83 patients suitable for analysis in this study. The study population is identical to our analysis regarding the influence of platelet counts on post-traumatic lung injury [37]. The ISS subgroup < 35 ranges from ISS 18–34, and the <60 years subgroup consists of patients between 20–51 years of age. For improved clarity, the terms ISS < 35 and age < 60 years are used.

Details of the study population are depicted in Table 1.

#### 3.1.2. Dynamics of Platelet Counts and Serum Creatinine Concentration

**Figure 1:**
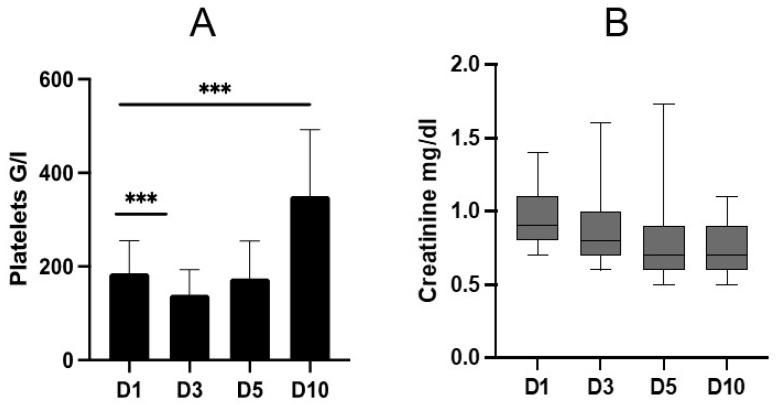
Dynamics of post-traumatic platelet counts (**A**) and serum creatinine concentration (**B**) on days (D) 1, 3, 5, and 10. Data are presented as mean and standard deviation (**A**) and median and interquartile range, and 10th to 90th percentile (**B**). A: mixed-effect analysis revealed a significant change in platelet count dynamics within the observation period (*p* ≤ 0.001). On D3, platelets decreased significantly compared to D1 (*p* ≤ 0.001). On D10, there was a significant increase compared to D1 (*p* ≤ 0.001). B: creatinine concentration remained stable without significant differences. *** = *p* < 0.001.

Platelet counts and serum creatinine concentration were gathered on the subsequent days until day 10 after admission (Figure 1).

Platelet counts were significantly decreased on day 3 compared to day 1 (D1: mean 185.5 G/L ± 69.6 G/L vs. D3: mean 139.9 G/L ± 53.5 G/L; *p* ≤ 0.001) and increased significantly on day 10 (D1: mean 185.5 G/L ± 69.6 G/L vs. D10: mean 350.9 G/L ± 142 G/L; *p* ≤ 0.001).

The dynamics of serum creatinine concentrations did not show any significant changes.

An additional table depicts platelet count and creatinine concentration on days 1, 3, 5, and 10 and a comparison of the respective days to day 1 (see Supplementary Table S1).

#### 3.1.3. Subgroup Analysis of Dynamics of Platelet Counts and Creatinine Concentration

**Figure 2:**
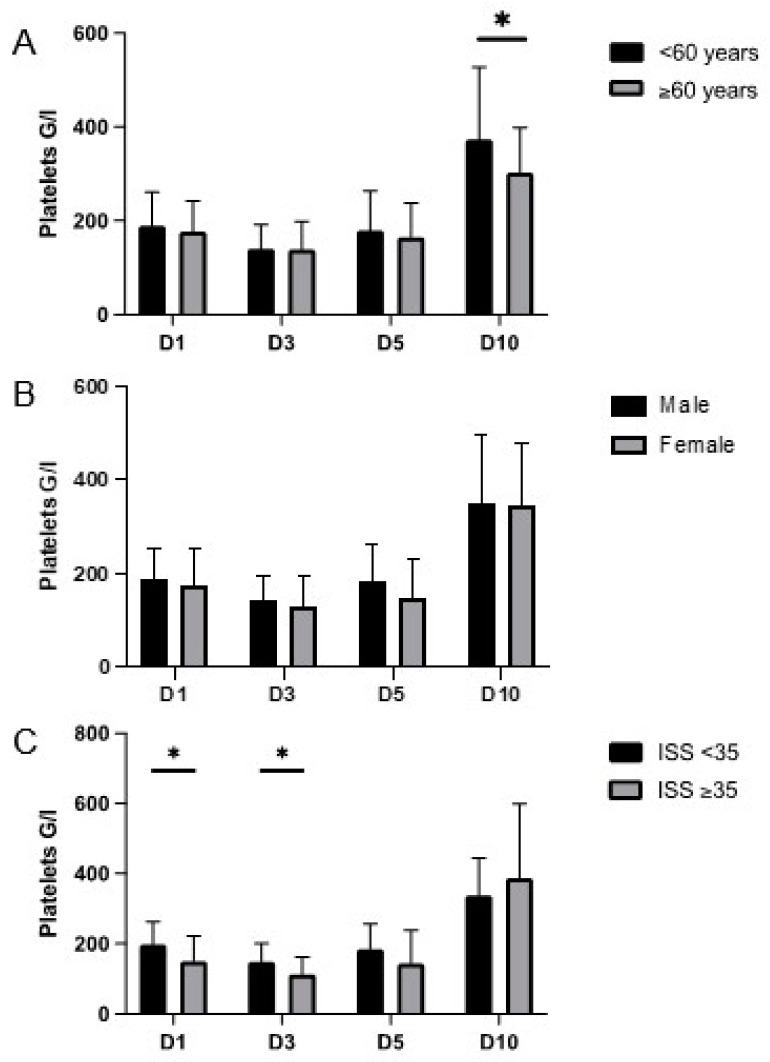
Platelet concentrations on days (D) 1, 3, 5, and 10 in the subgroups age (**A**), gender (**B**), and injury severity (**C**). Data are presented as mean and standard deviation. Concerning age, there was a significant increase in platelet counts in patients younger than 60 years compared to older individuals on D10 (*p* = 0.039). There were no significant differences between male and female patients. Platelet counts in patients with an ISS ≥ 35 were significantly decreased on D1 (*p* = 0.012) and D3 (*p* = 0.011) compared to patients with lower injury severity. * = *p* < 0.05.

Patients younger than 60 years showed a significantly increased platelet count on day 10 as compared to patients older than 60 years (<60 years: mean 373.2 G/L ± 154.8 G/L vs. ≥60 years: mean 302.8 G/L ± 96.1 G/L; *p* = 0.039) (Figure 2A). Sex did not affect platelet count following trauma-induced injury, and there was no significant difference in platelet count on the respective days between male and female patients (Figure 2B,C). Patients with an ISS less than 35 showed a significantly increased platelet count on day 1 (ISS < 35: mean 197.7 G/L ± 65.5 G/L vs. ISS ≥ 35: mean 150.6 G/L ± 70.9 G/L; *p* = 0.012) and day 3 (ISS < 35: mean 149 G/L ± 52.2 G/L vs. ISS ≥ 35: mean 113.2 G/L ± 49.4 G/L; *p* = 0.011) as compared to patients with a calculated ISS higher than 35. These results are reported and discussed in our previous investigation regarding the impact of platelets on lung physiology [37]. The extension of the observation period until day 10 did not reveal any new findings.

**Figure 3:**
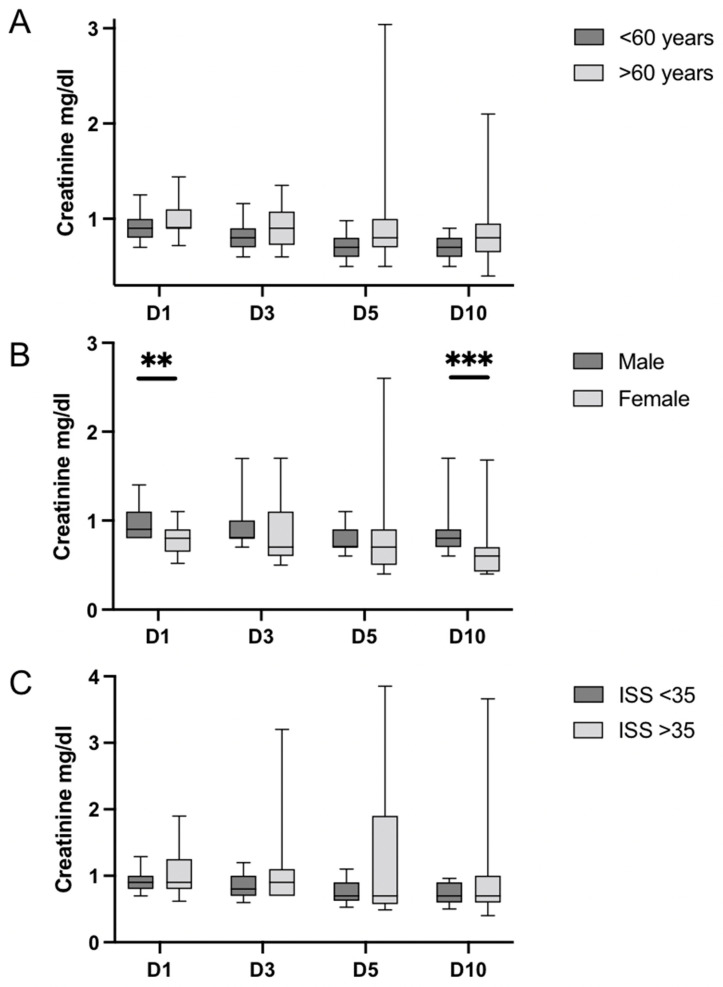
Serum creatinine concentration on days (D) 1, 3, 5, and 10 in the subgroups age (**A**), sex (**B**), and injury severity (**C**). Data are presented as median and interquartile range and 10th to 90th percentile. Overall dynamics of creatinine concentration show a stable trend within the respective subgroups. Regarding age, there were no significant differences. Male patients presented significantly increased creatinine concentrations compared to females on D1 (*p* = 0.003) and D10 (*p* ≤ 0.001). With regard to injury severity, there were no significant differences. IQR = interquartile range; ** = *p* < 0.01; *** = *p* < 0.001.

The comparison of the serum creatinine concentration revealed significant differences in the gender subgroups, but no differences were detected depending on age and injury severity (Figure 3A–C). Creatinine concentration was significantly increased in male patients on day 1 (male: median 0.9 IQR 0.8–1.1 vs. female: median 0.8 mg/dL IQR 0.7–0.9 mg/dL; *p* = 0.003) and day 10 (male: median 0.8 mg/dL IQR 0.7–0.9 mg/dL vs. female: 0.6 mg/dL IQR 0.4–0.7 mg/dL; *p* ≤ 0.001) as compared to female patients.

Additional tables depict platelet count (Supplementary Table S2) and creatinine concentration (Supplementary Table S3) within the respective subgroups on days 1, 3, 5, and 10 after trauma.

### 3.2. Correlation Analysis

In the second step of our data analysis, we performed a correlation analysis to identify the impact of platelet counts on post-traumatic serum creatinine concentration.

**Figure 4:**
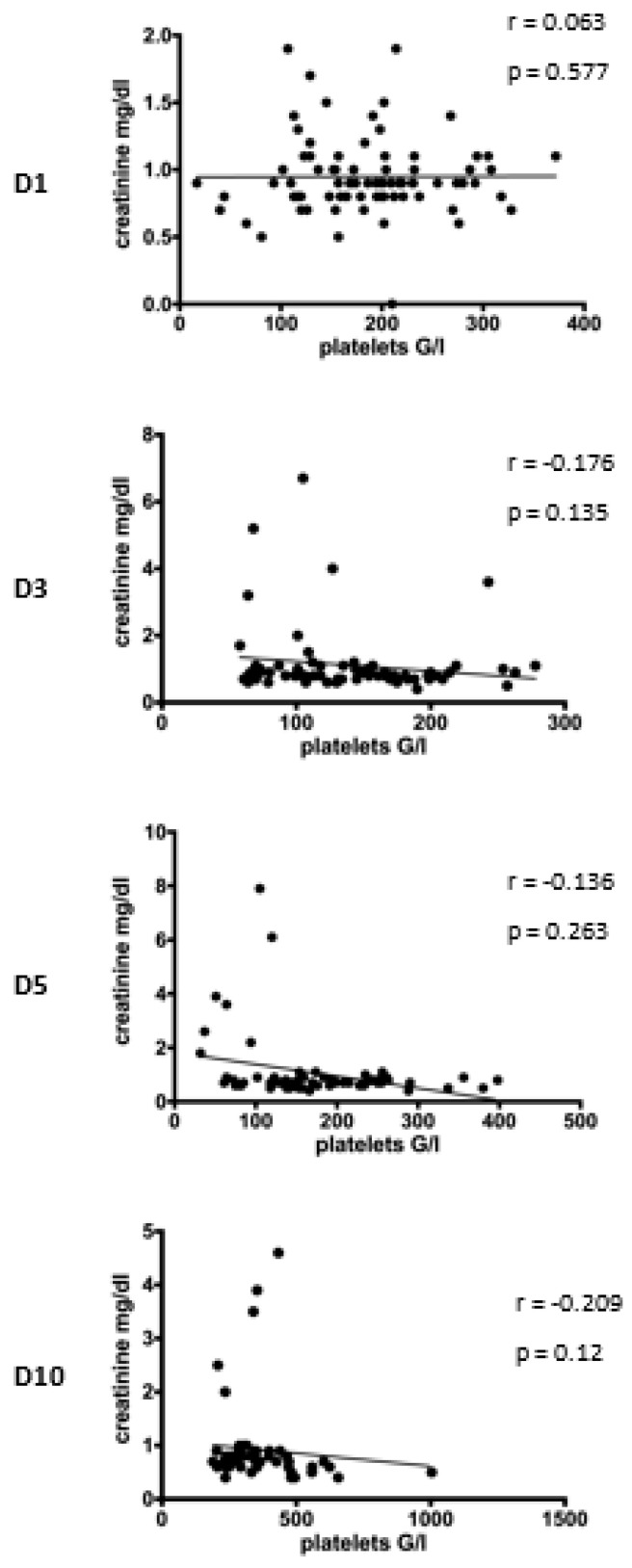
Correlation between platelet count and creatinine concentration following injury. Correlation analysis at D1 after admission reveals no correlation between platelet count and creatinine concentration (r = 0.063, *p* = 0.577). On the subsequent days, there is a slight trend that thrombocytosis is associated with decreased creatinine concentration (D3: r = −0.176, *p* = 0.135; D5: r = −0.136, *p* = 0.263; D10: r = −0.209, *p* = 0.12). D = day, r= Pearson/Spearman’s correlation coefficient, *p* = level of significance.

First, the correlation between platelet count and serum creatinine concentration was analyzed amongst all included patients (Figure 4). However, correlation analysis did not reach the level of significance.

In a second step, multiple least-squares linear regression analysis was performed to identify the potential influence of the epidemiological factors age, sex, and ISS (Table 2). To detect the impact on kidney function, serum creatinine concentration was set as a dependent variable, and analysis was performed for the subsequent days (days 1, 3, 5, and 10) after multiple trauma.

On day 1, advanced age (*p* = 0.01), male sex (*p* = 0.004) and severe injury (*p* = 0.014) were significantly associated with increasing creatinine concentration.

On day 5, platelet counts significantly correlated with a decrease in serum creatinine concentration (*p* = 0.046). There was no effect on day 3 and day 10.

## 4. Discussion

Recently, platelets have become of interest as contributing co-factors in post-traumatic hyperinflammation [13,16,43].

Our goal was to transfer the molecular understanding of the impact of platelets on AKI to a clinical setting [21,22,23].

We conducted a descriptive and correlation analysis of 83 patients who suffered multiple trauma to identify the influence of platelet counts on serum creatinine concentration as the parameter for post-traumatic kidney physiology.

### 4.1. Descriptive Analysis

#### 4.1.1. Dynamics of Platelet Count after Multiple Trauma

Compared to the baseline value on day 1, we observed a significant reduction of platelet counts on day 3 and a significant increase on day 10 after trauma. Changes in platelet concentrations within 72 h after trauma were described and discussed in our previous analysis regarding the influence of platelets on post-traumatic lung injury [37]. The extension of the observation period until day 10 revealed decreased platelet counts in older patients compared to younger patients.

Changes in post-traumatic dynamics of platelet counts depending on injury severity were also described by Hefele and coworkers [17]. They discovered an increase in platelet counts with impaired function to a maximum concentration on day 10 after trauma. Similar to our recent findings, platelet counts were increased in patients with minor injuries. This might be explained by blood loss or dilution effects caused by fluid and blood volume resuscitation in patients suffering more severe trauma. A decrease in platelet counts is also observed when platelets and neutrophils cumulate in damaged tissue (sequestration) and contribute to local inflammatory processes [29,31,44]. We observed that this effect diminishes over time (no difference on day 5 and day 10), which could be attributed to transfusion therapy and a beginning transition to a stabilized condition after damage control surgery.

Nydam and coworkers also described a connection between low platelet counts and increased ISS [33]. In line with our results, they discovered patients’ age as an influencing factor. Older patients presented lower platelet counts than younger patients. However, this effect was already present within the first 48 h, when we detected a significant difference ten days after trauma.

#### 4.1.2. Dynamics of Serum Creatinine Concentration after Multiple Trauma

Post-traumatic serum creatinine concentrations were slightly elevated until day 5 and decreased until day 10. Dynamic changes did not reach a level of significance.

Haines and coworkers also described decreasing creatinine concentrations on day 10 in patients with longer-lasting ICU stays [45]. Creatinine is an important substrate of muscle metabolism. Catabolic processes, such as muscle wasting during ICU, might be a potential theory to explain increasing concentrations after multiple trauma.

We observed significantly increased creatinine concentrations in men compared to women on day 1 and day 10 after admission. We believe sex-related differences in muscle volume to be responsible for this effect. The acute soft tissue trauma and muscle manipulation during early damage control surgery or later in definite care surgery could aggravate this effect.

### 4.2. Correlation Analysis of Platelet Count and Serum Creatinine Concentration after Multiple Trauma

The analysis of the entire study population revealed no significant correlation between platelet counts and serum creatinine concentration on subsequent days after multiple trauma.

Multiple linear regression analysis revealed an association between advanced age, male gender, injury severity, and increasing creatinine concentration within 24 h after multiple trauma, potentially indicating impairing kidney physiology.

On day 5, increasing platelet counts are associated with decreased creatinine concentration.

The general impact of platelets on overall MOF by participation in immuno-inflammatory processes after trauma is well investigated [8,38,39,46]. For example, Nydam and coworkers discovered that low post-traumatic platelet counts are an independent risk factor for developing MOF, whereas elevated platelet counts seem protective with a lower likelihood of mortality [33]. In line with our results, this effect was observed between day 3 and day 10. According to experimental findings of an ARDS model, this effect might be explained by platelet sequestration. Activated by proinflammatory mediators, platelets migrate from the bloodstream to organ tissue, interact with neutrophils and further promote inflammation and tissue damage [30,31,47,48]. We detected an association between platelet counts and an increase in serum creatinine concentrations on day 5 after multiple trauma, which supports the findings by Ciesla and coworkers, who report that during MOF development, immuno-inflammatory kidney impairment sets in around 6 days after initial trauma [49].

So far, the influence of platelets on kidney function in the setting of multiple trauma patients has not been the subject of previous studies. But there is evidence that platelets play a role in the pathophysiology of AKI in general.

Similar to post-traumatic ARDS pathophysiology, components are impaired endothelial control leading to increased platelet activation, stimulation of hemodynamic alterations, promotion of renal inflammation by leukocyte interaction, activation of the complement system, and cytokine release [50]. Platelets also became a potential target in AKI therapy. There is growing evidence for improved kidney function after administering antiplatelet agents [27,28,51].

On the other hand, Lax and coworkers demonstrated inhibition of inflammatory processes modulated by platelets after activation of the C-type-lectin-like receptor 2 (CLEC2) [52]. Taken together, platelets show proinflammatory but also protective effects in the pathogenesis of AKI.

We were able to present that the female gender potentially tends to positively influence post-traumatic kidney physiology early after trauma by being an independent factor for decreased creatinine concentration.

Potentially due to the protective effect of estradiol and its derivates, women tend to have a survival advantage over men [53,54,55]. There is also evidence that susceptibility to AKI is also less frequently observed in females [56]. Furthermore, AKI animal models demonstrated reduced inflammation and increased tubular cell regeneration after administration of estradiol, potentially induced by depressed neutrophil infiltration and modulation of inflammatory cytokines [57,58].

However, the unequal sex allocation of our study population could bias our observations.

Besides gender, advanced age and trauma severity also tend to predict increased serum creatinine concentrations after trauma. Our findings confirm the results of similar studies identifying age and ISS as independent risk factors for AKI [59,60,61]. In variation, we exclusively observed this effect within the first 24 h after trauma with no effect on the subsequent days. This rather identifies an early direct renal impact (e.g., shock, rhabdomyolysis, reperfusion injury) instead of delayed immunological influences to be responsible for increased creatinine concentrations in our collective.

### 4.3. Limitations

Although all attempts were made to ensure the accuracy of data, the retrospective character of the study limits the control of data quality. Based on the patient data sets, the assessment of creatinine levels was the only parameter to assess kidney physiology. We could not assess additional parameters such as urine output or urine balance to fulfill coexisting definitions for AKI [21,62,63]. Therefore, amongst others, changes in muscle metabolism and pre-traumatic kidney insufficiency-both affecting creatinine concentration-might bias our findings. Concerning platelet concentrations, we had no access to individual transfusion therapy history, which could bias our observations. In addition, trauma patients are an inconsistent study population due to variations in injury patterns and individualized therapies. Finally, a cohort of 83 individuals is too small for clear conclusions. However, future larger investigations could follow up on our results. Since statistical correlation does not inevitably indicate causation, every effort was made to discuss our findings with extreme caution.

## 5. Conclusions

For the first time, we investigated the direct influence of platelets on kidney physiology in multiple trauma patients. Following trauma, an increase in platelet concentration can be detected. The kinetics are influenced by age and trauma severity. Gender was identified as an influential factor for post-traumatic changes in creatinine concentration.

There was no clear correlation between platelet counts and serum creatinine concentration on subsequent days after multiple trauma.

Multiple linear regression analysis identified age, male gender, and injury severity as independent factors for increased creatinine concentration within 24 h after trauma. Platelets were associated with decreased creatinine concentration on day 5 after trauma, potentially indicating an immunological influence on post-traumatic kidney physiology. High platelet counts might correlate with renal integrity.

Our results add additional knowledge concerning the influence of platelets in multiple trauma patients, and might be used for future studies.

## Figures and Tables

**Table 1:** Characteristics of the study population.

	n = 83 (100%)	Mean ± SD/Median (IQR Q_25_–Q_75_)
**Age**		Median 51 years (34–64 years)
<60 years	57 (68.7%)	Median 43 years (29–51 years)
≥60 years	26 (31.3%)	Median 73 years (66–76 years)
**Gender**		
Men	62 (74.7%)	-
Women	21 (25.3%)	-
**Trauma Mechanism**		
Blunt	76 (91.6%)	-
Penetrating	7 (8.4%)	-
**Abbreviated Injury Scale**		
Head	59 (71.1%)	Median 3 (0–4)
face	28 (33.7%)	Median 0 (0–2)
thorax	45 (54.2%)	Median 2 (0–3)
abdomen	28 (33.7%)	Median 0 (0–3)
extremities /pelvis	62 (74.7%)	Median 3 (0–4)
other	25 (30.1%)	Median 0 (0–1)
**Injury Severity Score**	-	Median 22 (18–36)
<35	62 (74.7%)	Median 19 (17–25)
≥35	21 (25.3%)	Median 41 (38–57)
**Need for intensive care medicine**		
Duration	0–56 days	Mean 8.3 days ± 13.0 days
No ICU stay	16 (19%)	Mean 15.1 days ± 5.8 days
Admission to ICU	67 (81%)	Mean 10.1 days ± 13.7 days
**Outcome**		
Deceased	10 (12%)	-
Survivors	73 (88%)	-

SD = standard deviation, IQR = interquartile range, ICU = intensive care unit.

**Table 2:** Multiple linear regression with creatinine concentration as a dependent variable and platelet count, age, sex, injury severity, and length of ICU stay as independent variables on day (D) 1, 3, 5, and 10.

		Variable	Estimate	Standard Error	95% CI	*p*
**D1**	Creatinine (mg/dL)	Platelets	≤0.001	≤0.001	<−0.001 to ≤0.001	0.377
		Age	≤0.004	0.002	≤0.001 to 0.001	0.01 *
		Sex (Female)	−0.197	0.066	−0.328 to −0.066	0.004 **
		ISS	0.006	0.003	<0.001 to 0.012	0.014 *
		Days on ICU	−0.002	0.002	−0.007 to 0.003	0.361
**D3**	Creatinine (mg/dL)	Platelets	−0.002	0.002	−0.006 to 0.003	0.538
		Age	0.005	0.007	−0.008 to 0.018	0.451
		Sex (Female)	−0.371	0.283	−0.934 to 0.193	0.194
		ISS	0.015	0.012	−0.009 to 0.040	0.216
		Days on ICU	0.004	0.01	−0.016 to 0.025	0.657
**D5**	Creatinine (mg/dL)	Platelets	−0.004	0.001	−0.008 to −7.722	0.046 *
		Age	0.007	0.008	−0.009 to 0.0219	0.141
		Sex (Female)	−0.415	0.333	−1.080 to 0.250	0.139
		ISS	0.017	0.014	−0.011 to 0.045	0.147
		Days on ICU	−0.004	0.012	−0.028 to 0.019	0.044
**D10**	Creatinine (mg/dL)	Platelets	≤−0.001	≤0.001	−0.002 to 0.001	0.482
		Age	0.004	0.006	−0.009 to 0.017	0.515
		Sex (Female)	−0.268	0.244	−0.758 to 0.221	0.276
		ISS	0.016	0.011	−0.006 to 0.039	0.156
		Days on ICU	0.001	0.009	−0.016 to 0.019	0.882

ISS = injury severity score, ICU = intensive care unit, level of significance was set as *p* < 0.05. * = *p* < 0.05; ** = *p* < 0.01.

## Data Availability

The data is available on request due to the waiver of informed consent (see above).

## Supplementary Materials

The following supporting information can be downloaded at: https://www.mdpi.com/article/10.3390/medicina58070901/s1, Table S1: Platelet count and creatinine concentration on day (D) 1, 3, 5, and 10 of the entire study population.; Table S2: Platelet count on day (D) 1, 3, 5, and 10 dependent on age, gender, and injury severity.; Table S3: Creatinine concentration on day (D) 1, 3, 5, and 10 dependent on age, gender, and injury severity.

## References

[1] WHO The Top 10 Causes of Death. https://www.who.int/news-room/fact-sheets/detail/the-top-10-causes-of-death.

[2] Demetriades D., Kimbrell B., Salim A., Velmahos G., Rhee P., Preston C., Gruzinski G., Chan L. (2005). Trauma Deaths in a Mature Urban Trauma System: Is “Trimodal” Distribution a Valid Concept?. J. Am. Coll. Surg..

[3] Ciesla D.J., Moore E.E., Johnson J.L., Burch J.M., Cothren C.C., Sauaia A. (2005). A 12-Year Prospective Study of Postinjury Multiple Organ Failure: Has anything changed?. Arch. Surg..

[4] Sauaia A., Moore E.E., Johnson J.L., Chin T.L., Banerjee A., Sperry J.L., Maier R.V., Burlew C.C. (2014). Temporal trends of postinjury multiple-organ failure: Still resource intensive, morbid, and lethal. J. Trauma Acute Care Surg..

[5] Moore F.A., Moore E.E. (1995). Evolving Concepts in the Pathogenesis of Postinjury Multiple Organ Failure. Surg. Clin. N. Am..

[6] Zedler S., Faist E. (2006). The impact of endogenous triggers on trauma-associated inflammation. Curr. Opin. Crit. Care.

[7] Gentile L.F., Cuenca A.G., Efron P.A., Ang D., Bihorac A., McKinley B.A., Moldawer L.L., Moore F.A. (2012). Persistent inflammation and immunosuppression: A common syndrome and new horizon for surgical intensive care. J. Trauma Acute Care Surg..

[8] Sauaia A., Moore F.A., Moore E.E. (2017). Postinjury Inflammation and Organ Dysfunction. Crit. Care Clin..

[9] Nurden A.T. (2011). Platelets, inflammation and tissue regeneration. Thromb. Haemost..

[10] Bergmann C.B., Hefele F., Unger M., Huber-Wagner S., Biberthaler P., Van Griensven M., Hanschen M. (2016). Platelets modulate the immune response following trauma by interaction with CD4+ T regulatory cells in a mouse model. Immunol. Res..

[11] Bock M., Bergmann C.B., Jung S., Kalbitz M., Relja B., Huber-Wagner S., Biberthaler P., van Griensven M., Hanschen M. (2018). The posttraumatic activation of CD4+ T regulatory cells is modulated by TNFR2- and TLR4-dependent pathways, but not by IL-10. Cell. Immunol..

[12] Klinger M.H., Jelkmann W. (2002). Review: Role of Blood Platelets in Infection and Inflammation. J. Interferon Cytokine Res..

[13] Morrell C.N., Aggrey A.A., Chapman L.M., Modjeski K.L. (2014). Emerging roles for platelets as immune and inflammatory cells. Blood.

[14] Zarbock A., Polanowska-Grabowska R.K., Ley K. (2007). Platelet-neutrophil-interactions: Linking hemostasis and inflammation. Blood Rev..

[15] Elzey B.D., Sprague D.L., Ratliff T.L. (2005). The emerging role of platelets in adaptive immunity. Cell. Immunol..

[16] Henn V., Slupsky J.R., Gräfe M., Anagnostopoulos I., Forster R., Müller-Berghaus G., Kroczek R.A. (1998). CD40 ligand on activated platelets triggers an inflammatory reaction of endothelial cells. Nature.

[17] Hefele F., Ditsch A., Krysiak N., Caldwell C.C., Biberthaler P., van Griensven M., Huber-Wagner S., Hanschen M. (2019). Trauma Induces Interleukin-17A Expression on Th17 Cells and CD4+ Regulatory T Cells as Well as Platelet Dysfunction. Front. Immunol..

[18] Veer C.V., Van Der Poll T., De Stoppelaar S.F. (2014). The role of platelets in sepsis. Thromb. Haemost..

[19] Zarbock A., Gomez H., Kellum J.A. (2014). Sepsis-induced acute kidney injury revisited: Pathophysiology, prevention and future therapies. Curr. Opin. Crit. Care.

[20] Bellomo R., Kellum A.J., Ronco C. (2012). Acute kidney injury. Lancet.

[21] Brandt M.-M., Falvo A.J., Rubinfeld I.S., Blyden D., Durrani N.K., Horst H.M. (2007). Renal Dysfunction in Trauma: Even a Little Costs a Lot. J. Trauma Acute Care Surg..

[22] Morris J.A., Mucha P., Ross S.E., Moore B.F.A., Hoyt D.B., Gentilello L., Landercasper J., Feliciano D.V., Shackford S.R. (1991). Acute Posttraumatic Renal Failure: A Multicenter Perspective. J. Trauma.

[23] Podoll A.S., Kozar R., Holcomb J.B., Finkel K.W. (2013). Incidence and Outcome of Early Acute Kidney Injury in Critically-Ill Trauma Patients. PLoS ONE.

[24] Schrier R.W., Wang W. (2004). Acute Renal Failure and Sepsis. N. Engl. J. Med..

[25] Singbartl K., Forlow S.B., Ley K. (2001). Platelet, but not endothelial, P-selectin is critical for neutrophil-mediated acute postischemic renal failure. FASEB J..

[26] Singbartl K., Ley K. (2004). Leukocyte recruitment and acute renal failure. J. Mol. Med..

[27] Jansen M.P., Emal D., Teske G.J., Dessing M.C., Florquin S., Roelofs J.J. (2017). Release of extracellular DNA influences renal ischemia reperfusion injury by platelet activation and formation of neutrophil extracellular traps. Kidney Int..

[28] Hu H., Batteux F., Chéreau C., Kavian N., Marut W., Gobeaux C., Borderie D., Dinh-Xuan A.T., Weill B., Nicco C. (2011). Clopidogrel protects from cell apoptosis and oxidative damage in a mouse model of renal ischaemia-reperfusion injury. J. Pathol..

[29] Zarbock A., Singbartl K., Ley K. (2006). Complete reversal of acid-induced acute lung injury by blocking of platelet-neutrophil aggregation. J. Clin. Investig..

[30] Zarbock A. (2009). The role of platelets in acute lung injury (ALI). Front. Biosci..

[31] Looney M.R., Nguyen J.X., Hu Y., Van Ziffle J.A., Lowell C.A., Matthay M.A. (2009). Platelet depletion and aspirin treatment protect mice in a two-event model of transfusion-related acute lung injury. J. Clin. Investig..

[32] Kasotakis G., Starr N., Nelson E., Sarkar B., Burke P.A., Remick D.G., Tompkins R.G. (2018). The Inflammation and Host Response to Injury Investigators Platelet transfusion increases risk for acute respiratory distress syndrome in non-massively transfused blunt trauma patients. Eur. J. Trauma Emerg. Surg..

[33] Nydam T.L., Kashuk J.L., Moore E.E., Johnson J.L., Burlew C.C., Biffl W.L., Barnett C.C., Sauaia A. (2011). Refractory Postinjury Thrombocytopenia Is Associated with Multiple Organ Failure and Adverse Outcomes. J. Trauma Acute Care Surg..

[34] Howard B.M., Kornblith L.Z., Hendrickson C.M., Redick B.J., Conroy A.S., Nelson M.F., Callcut R.A., Calfee C.S., Cohen M.J. (2015). Differences in degree, differences in kind: Characterizing lung injury in trauma. J. Trauma Acute Care Surg..

[35] Harr J.N., Moore E.E., Johnson J., Chin T.L., Wohlauer M.V., Maier R., Cuschieri J., Sperry J., Banerjee A., Silliman C.C. (2013). Antiplatelet Therapy Is Associated with Decreased Transfusion-Associated Risk of Lung Dysfunction, Multiple Organ Failure, and Mortality in Trauma Patients. Crit. Care Med..

[36] Boyle A.J., Di Gangi S., Hamid U.I., Mottram L.-J., McNamee L., White G., Cross L.M., McNamee J.J., O’Kane C.M., McAuley D.F. (2015). Aspirin therapy in patients with acute respiratory distress syndrome (ARDS) is associated with reduced intensive care unit mortality: A prospective analysis. Crit. Care.

[37] Greve F., Mair O., Aulbach I., Biberthaler P., Hanschen M. (2022). Correlation between Platelet Count and Lung Dysfunction in Multiple Trauma Patients—A Retrospective Cohort Analysis. J. Clin. Med..

[38] Sauaia A., Moore F.A., Moore E.E., Haenel J.B., Read R.A., Lezotte D.C. (1994). Early Predictors of Postinjury Multiple Organ Failure. Arch. Surg..

[39] Sauaia A., Moore F.A., Moore E.E., Norris J.M., Lezotte D.C., Hamman R.F. (1998). Multiple Organ Failure Can Be Predicted as Early as 12 Hours after Injury. J. Trauma Acute Care Surg..

[40] Bösch F., Angele M.K., Chaudry I.H. (2018). Gender differences in trauma, shock and sepsis. Mil. Med. Res..

[41] Gennarelli T., Wodzin E. (2016). Abbreviated Injury Scale 2005: Update 2008. https://www.aaam.org/how-do-i-cite-the-ais-dictionary/.

[42] Baker S.P., O’Neill B., Haddon W., Long W.B. (1974). The injury severity score: A method for describing patients with multiple injuries and evaluating emergency care. J. Trauma.

[43] Boilard E., Nigrovic P.A., Larabee K., Watts G.F.M., Coblyn J.S., Weinblatt M.E., Massarotti E.M., Remold-O’Donnell E., Farndale R.W., Ware J. (2010). Platelets Amplify Inflammation in Arthritis via Collagen-Dependent Microparticle Production. Science.

[44] Laschke M.W., Dold S., Menger M.D., Jeppsson B., Thorlacius H. (2008). Platelet-dependent accumulation of leukocytes in sinusoids mediates hepatocellular damage in bile duct ligation-induced cholestasis. J. Cereb. Blood Flow Metab..

[45] Haines R.W., Zolfaghari P., Wan Y., Pearse R.M., Puthucheary Z., Prowle J.R. (2019). Elevated urea-to-creatinine ratio provides a biochemical signature of muscle catabolism and persistent critical illness after major trauma. Intensiv. Care Med..

[46] Sauaia A., Moore F.A., Moore E.E., Lezotte D.C. (1996). Early Risk Factors for Postinjury Multiple Organ Failure. World J. Surg..

[47] Bhatia M., Moochhala S. (2004). Role of inflammatory mediators in the pathophysiology of acute respiratory distress syndrome. J. Pathol..

[48] Gaertner F., Ahmad Z., Rosenberger G., Fan S., Nicolai L., Busch B., Yavuz G., Luckner M., Ishikawa-Ankerhold H., Hennel R. (2017). Migrating Platelets Are Mechano-scavengers that Collect and Bundle Bacteria. Cell.

[49] Ciesla D.J., Moore E.E., Johnson J.L., Burch J.M., Cothren C.C., Sauaia A. (2005). The role of the lung in postinjury multiple organ failure. Surgery.

[50] Jansen M.P.B., Florquin S., Roelofs J.J.T.H. (2018). The role of platelets in acute kidney injury. Nat. Rev. Nephrol..

[51] Ragab D., Abdallah D.M., El-Abhar H.S. (2014). Cilostazol Renoprotective Effect: Modulation of PPAR-γ, NGAL, KIM-1 and IL-18 Underlies Its Novel Effect in a Model of Ischemia-Reperfusion. PLoS ONE.

[52] Lax S., Rayes J., Wichaiyo S., Haining E.J., Lowe K., Grygielska B., Laloo R., Flodby P., Borok Z., Crandall E.D. (2017). Platelet CLEC-2 protects against lung injury via effects of its ligand podoplanin on inflammatory alveolar macrophages in the mouse. Am. J. Physiol. Cell. Mol. Physiol..

[53] Frink M., Pape H.-C., van Griensven M., Krettek C., Chaudry I.H., Hildebrand F. (2007). Influence of sex and age on mods and cytokines after multiple injuries. Shock.

[54] Mostafa G., Huynh T., Sing R.F., Miles W.S., Norton H.J., Thomason M.H. (2002). Gender-Related Outcomes in Trauma. J. Trauma Acute Care Surg..

[55] Wohltmann C.D., Franklin G.A., Boaz P.W., Luchette F.A., Kearney P.A., Richardson J., Spain D.A. (2001). A multicenter evaluation of whether gender dimorphism affects survival after trauma. Am. J. Surg..

[56] Kang K.P., Lee J.E., Lee A.S., Jung Y.J., Kim D., Lee S., Hwang H.P., Kim W., Park S.K. (2014). Effect of gender differences on the regulation of renal ischemia-reperfusion-induced inflammation in mice. Mol. Med. Rep..

[57] Wu C.-C., Chang C.-Y., Chang S.-T., Chen S.-H. (2016). 17β-Estradiol Accelerated Renal Tubule Regeneration in Male Rats after Ischemia/Reperfusion-Induced Acute Kidney Injury. Shock.

[58] Kasımay O., Şener G., Çakır B., Yüksel M., Çetinel S., Contuk G., Yeğen B. (2009). Estrogen Protects against Oxidative Multiorgan Damage in Rats with Chronic Renal Failure. Ren. Fail..

[59] Perkins Z.B., Captur G., Bird R., Gleeson L., Singer B., O’Brien B. (2019). Trauma induced acute kidney injury. PLoS ONE.

[60] Eriksson M., Brattström O., Mårtensson J., Larsson E., Oldner A. (2015). Acute kidney injury following severe trauma: Risk factors and long-term outcome. J. Trauma Acute Care Surg..

[61] Haines R.W., Lin S.-P., Hewson R., Kirwan C.J., Torrance H.D., O’Dwyer M.J., West A., Brohi K., Pearse R., Zolfaghari P. (2018). Acute Kidney Injury in Trauma Patients Admitted to Critical Care: Development and Validation of a Diagnostic Prediction Model. Sci. Rep..

[62] Bellomo R., Ronco C., Kellum J.A., Mehta R.L., Palevsky P., Acute Dialysis Quality Initiative Workgroup (2004). Acute renal failure—Definition, outcome measures, animal models, fluid therapy and information technology needs: The Second International Consensus Conference of the Acute Dialysis Quality Initiative (ADQI) Group. Crit. Care.

[63] Mehta R.L., Kellum J.A., Shah S.V., Molitoris B.A., Ronco C., Warnock D.G., Levin A., Acute Kidney Injury Network (2007). Acute Kidney Injury Network: Report of an initiative to improve outcomes in acute kidney injury. Crit. Care.

