# Supplementary material for: The Clinical Impact of Platelets on Post-Injury Serum Creatinine Concentration in Multiple Trauma Patients: A Retrospective Cohort Study
Source: Medicina (Kaunas). 2022 Jul 6;58(7):901. doi: 10.3390/medicina58070901 (PMC9317692; doi:10.3390/medicina58070901)
Supplement: Supplementary file 1 [file medicina-58-00901-s001.zip › medicina-1738415-supplementary.pdf]

Supplementary Table S1. Platelet count and creatinine concentration on day (D) 1, 3, 5 and 10 of the entire study population.

	**Platelet count (G/l) (Mean ± SD)**	**P**	**Creatinine (mg/dl) (Mean ± SD)**	**P**
**D1**	185.5 ± 69.6	-	1 ± 0.3	
**D3**	139.9 ± 53.5	≤0.001***	1.1 ± 1	0.066
**D5**	174.6 ± 79.8	0.37	1.1 ± 1.2	≤0.001***
**D10**	350.9 ± 142	≤0.001***	0.9 ± 0.8	≤0.001***

Testing for statistical significance was performed between the respective days and D1 as base value during admission in the trauma bay. SD=standard deviation; ***=p<0.001 level of significance was set as p<0.05.

Supplementary Table S2. Platelet count on day (D) 1, 3, 5 and 10 in the subgroups age, sex and injury severity.

Platelet count (G/l)	<60 years (Mean ± SD)	≥60 years (Mean ± SD)	P
**D1**	189.4 ± 71.7	176.7 ± 65.3	0.437
**D3**	140.5 ± 50.5	138.7 ± 60.2	0.895
**D5**	179.5 ± 83.7	165.4 ± 72.6	0.460
**D10**	373.2 ± 154.8	302.8 ± 154.8	0.039*

	Male	Female	P
**D1**	188.6 ± 67.7	176.4 ± 76.0	0.518
**D3**	143.3 ± 49.8	129.8 ± 63.6	0.408
**D5**	183.9 ± 78.2	148.6 ± 80.6	0.109
**D10**	352.2 ± 146.7	347.3 ± 132.6	0.891

	ISS <35	ISS ≥35	P
**D1**	197.7 ± 65.5	150.6 ± 70.9	0.012*
**D3**	149.0 ± 52.2	113.2 ± 49.4	0.011*
**D5**	184.9 ± 72.3	143.7 ± 94.7	0.105
**D10**	337.5 ± 106.8	387.7 ± 211.2	0.694

SD=standard deviation; ISS=injury severity score; *=p<0.05; level of significance was set as p<0.05.

Supplementary Table S3. Creatinine concentration on day (D) 1, 3, 5 and 10 in the subgroups age, sex and injury severity.

**Creatinine (mg/dl)**	**<60 years (Mean ± SD)**	**≥60 years (Mean ± SD)**	**P**
**D1**	0.9 ± 0.3	1.0 ± 0.3	0.093
**D3**	1.2 ± 1.2	0.9 ± 0.3	0.360
**D5**	1.0 ± 1.3	1.1 ± 0.9	0.213
**D10**	0.9 ± 0.9	0.9 ± 0.5	0.176

	**Male**	**Female**	**P**
**D1**	1.0 ± 0.2	0.8 ± 0.3	0.003**
**D3**	1.2 ± 1.1	0.9 ± 0.6	0.069
**D5**	1.1 ± 1.3	1.0 ± 0.8	0.072
**D10**	1.0 ± 0.8	0.8 ± 0.7	≤0.001***

	**ISS <35**	**ISS ≥35**	**P**
**D1**	0.9 ± 0.2	1.0 ± 0.4	0.877
**D3**	1.1 ± 1.0	1.3 ± 1.1	0.675
**D5**	0.9 ± 1.1	1.4 ± 1.5	0.992
**D10**	0.8 ± 0.6	1.2 ± 1.1	0.827

SD=standard deviation; ISS=injury severity score; **=p<0.01; ***=p<0.001; level of significance was set as p<0.05.
